# Deep pain sensitivity is correlated with oral-health-related quality of life but not with prosthetic factors in complete denture wearers

**DOI:** 10.1590/1678-775720150174

**Published:** 2015

**Authors:** Yuri Martins COSTA, André Luís PORPORATTI, Priscila Brenner HILGENBERG-SYDNEY, Leonardo Rigoldi BONJARDIM, Paulo César Rodrigues CONTI

**Affiliations:** 1 - Universidade de São Paulo, Faculdade de Odontologia de Bauru, Departamento de Prótese, Bauru, SP, Brasil;; Aarhus University, Department of Dentistry, Section of Orofacial Pain and Jaw Function, Aarhus, Denmark.; 2 - Universidade de São Paulo, Faculdade de Odontologia de Bauru, Departamento de Prótese, Bauru, SP, Brasil.; 3 - Universidade de São Paulo, Faculdade de Odontologia de Bauru, Departamento de Ciências Biologicas, Bauru, SP, Brasil.

**Keywords:** Complete denture, Masticatory muscles, Pain threshold, Quality of life

## Abstract

Low pressure Pain Threshold (PPT) is considered a risk factor for Temporomandibular Disorders (TMD) and is influenced by psychological variables.

**Objectives:**

To correlate deep pain sensitivity of masticatory muscles with prosthetic factors and Oral-Health-Related Quality of Life (OHRQoL) in completely edentulous subjects.

**Material and Methods:**

A total of 29 complete denture wearers were recruited. The variables were: a) Pressure Pain Threshold (PPT) of the masseter and temporalis; b) retention, stability, and tooth wear of dentures; c) Vertical Dimension of Occlusion (VDO); d) Oral Health Impact Profile (OHIP) adapted to orofacial pain. The Kolmogorov-Smirnov test, the Pearson Product-Moment correlation coefficient, the Spearman Rank correlation coefficient, the Point-Biserial correlation coefficient, and the Bonferroni correction (α=1%) were applied to the data.

**Results:**

The mean age (standard deviation) of the participants was of 70.1 years (9.5) and 82% of them were females. There were no significant correlations with prosthetic factors, but significant negative correlations were found between the OHIP and the PPT of the anterior temporalis (r=-0.50, 95% CI-0.73 to 0.17, p=0.005).

**Discussion:**

The deep pain sensitivity of masticatory muscles in complete dentures wearers is associated with OHRQoL, but not with prosthetic factors.

## INTRODUCTION

Edentulism is highly prevalent in developing countries. In particular, 63% of the Brazilian elderly population wear complete dentures, and approximately 12% of the entire population is edentulous^5^. This warrants the need for a closer attention to this type of patient. The use of a complete denture is associated with impaired masticatory function, poor quality of life, and with oral lesions when the denture is inadequate^8^ ^,^ ^12^ ^,^ ^28^. However, the association with Temporomandibular Disorders (TMD) is tenuous; there is little solid evidence to the fact that edentulism is associated with TMD^22^. It is important to note that TMD is an umbrella term that encompasses different clinical conditions affecting the Temporomandibular Joint (TMJ) and masticatory muscles^21^. Although the signs and symptoms of TMD are not necessarily risk factors for TMD onset, TMJ noises and masticatory muscle pain were the most prevalent in a sample of elderly Brazilians^20^.

A recently published cross-sectional study revealed the wearing of complete dentures, or the poor prosthetic aspect of these dentures, significantly increases the prevalence of muscle pain on palpation^22^. Other studies have asserted the renewal of dentures with poor retention and stability reduces TMD symptomatology^1^. Finally, the incorrect Vertical Dimension of Occlusion (VDO) is historically associated with TMD^14^. Not only some mechanical/occlusal aspects are related to TMD signs or symptoms, but the biopsychosocial model for TMD development, as opposed to mechanistic concepts, has also been widely accepted^16^.

The Pressure Pain Threshold (PPT) can be defined as the minimum intensity of a pressure perceived as painful. It is an accurate and valid method to measure deep pain sensitivity^25^. The outcome of a PPT test is very informative in terms of deciphering the pathophysiological mechanisms of pain perception, particularly when this test is associated with a comprehensive somatosensory assessment. Inasmuch as a low PPT is considered a risk factor for the onset of TMD^23^, it would be of valuable interest to determine whether any prosthetic aspect of complete dentures wearers can, in fact, be correlated with PPT values. In addition to these mechanical and structural aspects, it is also important to evaluate the influence of Oral-Health-Related Quality of Life (OHRQoL) on the PPT of masticatory muscles, since psychological variables could play a role in the perception of muscle pain and in the variation of the PPT values^27^.

Based on the aforementioned, this study aimed to correlate the PPT values of the masticatory muscles with (1) retention, (2) stability, (3) tooth wear of dentures, (4) VDO, and (5) OHRQoL in completely edentulous subjects. The authors hypothesized *a priori* that there would be a correlation among PPT values versus prosthetic factors (VDO, retention, stability and tooth wear) and versus the OHRQoL.

## MATERIAL AND METHODS

### Design and ethics

This cross-sectional pilot study was conducted in accordance with the Declaration of Helsinki, and was approved by a local Human Research Ethics Committee.

### Subjects and recruitment

The eligible subjects included complete edentulous patients of both genders, who sought prosthetic treatment from 2010 to 2011. The inclusion criteria were: a) older than 50 years of age; b) wearing maxillary and mandibular dentures for at least one year. The exclusion criteria were: a) history of facial trauma or craniofacial surgical procedures; b) oral lesions, xerostomia or other oral manifestations of systematic diseases, and neurological disorders; c) painful TMD ongoing or in the last 30 days; d) previous treatments performed in the last 6 months for TMD. Only one expert in prosthodontics and orofacial pain performed the clinical examination to assess eligibility. The study included 29 subjects who fulfilled the above criteria. The informed consent was obtained from each subjects selected, after fully explaining the study aims and procedures.

### Variables

The PPT, the quality of the dentures (retention, stability and tooth wear), the VDO and the OHRQoL were assessed. The measurements were performed by a different examiner, who was also an expert in prosthodontics and orofacial pain and blinded to the clinical assessment.

### Deep Pain

**Figure 1:**
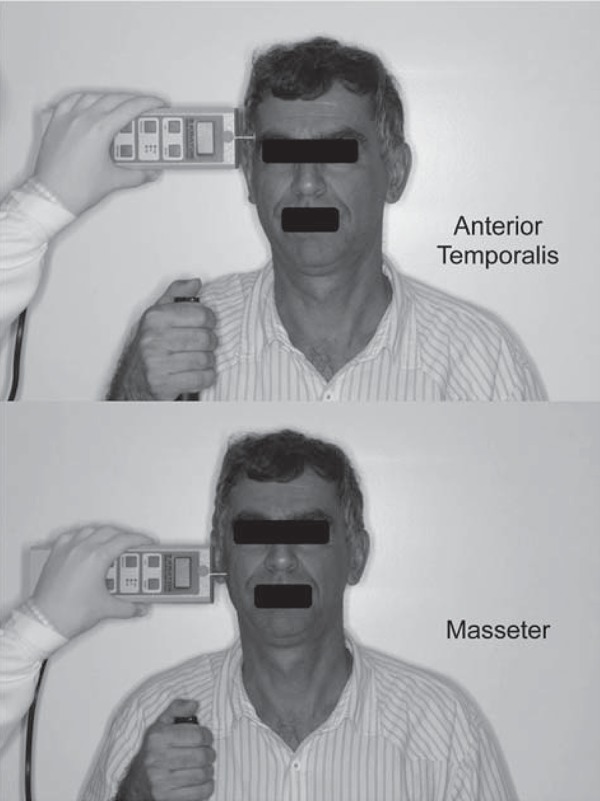
Illustration of the pressure pain threshold (PPT) assessment of the temporalis and masseter muscle

The PPT was determined with a digital dynamometer (KRATOS^®^, Cotia, SP, Brazil), used to record both sides of the masseter (body) and of the temporalis muscle (anterior, middle, and posterior belly) (Figure 1). This device has a circular flat tip (1 cm^2^) that was used to apply pressure with an application ratio of 0.5 kg/cm^2^/s. During the examination, the head of the subjects was firmly supported by the hand of the examiner. The subjects were informed that the objective was to measure their pain threshold, and not their tolerance. The device also has a button controlled by the subject, who was asked to press it the instant a pain sensation was felt; at which time the pressure was stopped, the value was displayed and recorded. Each site was randomly measured twice, with a 5-minute rest interval between the readings. The final PPT values comprised the mean of 2 trials made on both left and right sides.

### Denture quality

The retention of the dentures was rated as satisfactory (slight to moderate resistance to vertical pull, and little resistance to lateral forces) or unsatisfactory (no resistance to vertical pull and lateral forces)^3^. Similarly, stability was classified as satisfactory (no more than slight horizontal displacement or rotation movements when under pressure) or unsatisfactory (severe horizontal displacement or rotation movements when under pressure)^3^. Tooth wear was scored subjectively as follows: 0=no wear, 1=slight wear and 2=severe wear.

### Vertical Dimension of Occlusion (VDO)

The VDO was assessed quantitatively (VDO_quant_) by the Willis Gauge (vertical measurement from the subnasal region to the lower border of the chin, with the teeth in occlusion). In addition, the quality (VDO_quali_) was considered adequate or inadequate, based on the following techniques: facial measurement (Willis Method), phonetics and esthetic appearance^2^.

### Oral-Health-Related Quality of Life (OHRQoL)

The influence of the oral condition on quality of life was assessed using the Oral Health Impact Profile (OHIP) questionnaire adapted for orofacial pain patients^17^. This version is a short form of the original OHIP-49, which was validated for Brazilian Portuguese and showed good psychometrics (Cronbach’s alpha=0.963)^18^. The subjects were asked to score how often they experienced the impact of 30 statements in the last 6 months, based on a 5-point ordinal scale (0-4). Accordingly, the OHIP scores could range from 0 to 150, in which the higher scores indicated poor quality of life. Moreover, the questions and scores can be categorized into 6 dimensions: functional limitation, psychological discomfort, physical disability, psychological disability, social disability, and handicap.

### Statistical analysis

Quantitative variables (age, PPT, OHIP, and VDO_quant_) were expressed as means and as Standard Deviation (SD), and included a description of the gender distribution, denture retention, stability, tooth wear, and VDO_quali_. All quantitative variables were assessed for normal distribution, using the Kolmogorov-Smirnov test before performing the inferential analysis. The log_10_ transformation was applied in cases of abnormal distribution of the quantitative variables, i.e., social disability and handicap dimensions of the OHIP.

The Pearson Product-Moment Correlation Coefficient was used to correlate PPT values with VDO_quant_ and OHIP (total score and the 6 dimensions). Similarly, the Spearman Rank Correlation Coefficient was used to correlate PPT values and denture tooth wear. Lastly, the point-biserial correlation coefficient was used to correlate PPT values with denture retention and stability, and VDO_quali_. The magnitude of each effect measured was based on the r coefficient, and was scored as a small (r=0.3), moderate (r=0.5), or strong (r=0.7) correlation along with the 95% confidence interval (CI). The sample size in this study was considered too small to use regression models. In this respect, the problem of multiple comparisons was averted by applying a Bonferroni correction, lowering the significance level to 1% (α=0.01) as the cut-off point to determine the statistical significance.

## RESULTS

The clinical and demographic characteristics of the sample are detailed in Table 1. Mean age (SD) was 70.1 years (9.5) and 82% of the patients were females. The mean (SD) of the PPT values (kgf/cm^2^) for all three bellies (anterior, middle, and posterior) of the temporalis and for the masseter muscles were 1.64 (0.48), 1.90 (0.66), 2.13 (0.72), and 1.47 (0.52), respectively. The distribution of the maxillary dentures quality was similar, with satisfactory retention (55%) and stability (48%). Most of the mandibular dentures had unsatisfactory retention (82%) and stability (79%). Twenty-four percent of the maxillary and 20% of the mandibular dentures had severe tooth wear. The mean (SD) of the VDO in mm was 47.3 (8.0), and was considered adequate (VDO_quali_) in 58% of the patients. Finally, the mean (SD) of the OHIP was 20.82 (20.28).

**Table 1:** Demographic and clinical characteristics of the sample

Variables	Mean (SD) or N (%)
**Age (years)**	70.1 (9.5)
**Gender**	
Male	5 (17.2%)
Female	24 (82.7%)
**Pressure Pain Threshold (kgf/cm2)**	
Anterior Temporalis	1.64 (0.48)
Middle Temporalis	1.90 (0.66)
Posterior Temporalis	2.13 (0.72)
Masseter	1.47 (0.52)
**Quality of dentures (Maxillary)**	
Retention	
Satisfactory	16 (55.1%)
Unsatisfactory	13 (44.8%)
Stability	
Satisfactory	14 (48.2%)
Unsatisfactory	15 (51.7%)
Tooth wear	
No wear	12 (41.3%)
Loss of crown height ≤ 1/3	10 (34.4%)
Loss of crown height >1/3	7 (24.1%)
**Quality of dentures (Mandibular)**	
Retention	
Satisfactory	5 (17.2%)
Unsatisfactory	29 (82.7%)
Stability	
Satisfactory	6 (20.6%)
Unsatisfactory	23 (79.3%)
Tooth wear	
No wear	12 (41.3%)
Loss of crown height ≤ 1/3	11 (37.9%)
Loss of crown height >1/3	6 (20.6%)
**Vertical Dimension of Occlusion (VDO)**	
Quantitative (mm)	47.3 (8.0%)
Qualitative	
Adequate	17 (58.6%)
Inadequate	29 (41.3%)
**Oral Health Impact Profile (OHIP) - Total**	20.82 (20.28)
Functional limitation	3.44 (3.22)
Psychological discomfort	5.51 (5.12)
Physical disability	5.29 (4.68)
Psychological disability	3.29 (4.12)
Social disability	2.0 (4.2)
Handicap	1.22 (2.60)

SD=Standard Deviation

No significant correlations were found between the PPT values and denture quality or VDO_quant_/_quali_ after performing the Bonferroni correction (p>0.01). However, the correlation was significant, when considering the standard significance level (p<0.05) between tooth wear and PPT values of the anterior temporalis (maxillary denture, r= -0.38, 95% CI -0.66 to -0.01, p=0.03; mandibular denture, r=-0.38, 95% CI -0.66 to -0.01, p=0.03).

**Figure 2:**
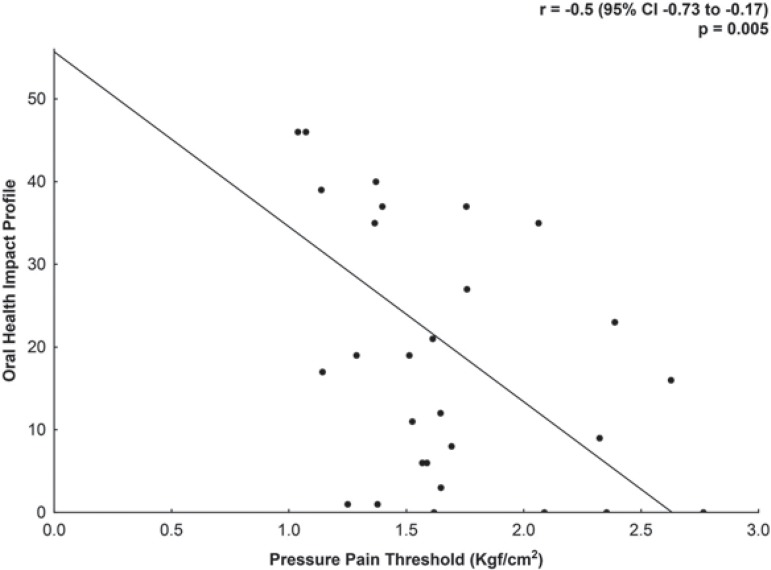
Correlation between pressure pain threshold (PPT) of the anterior temporalis and the Oral Health Impact Profile

**Figure 3:**
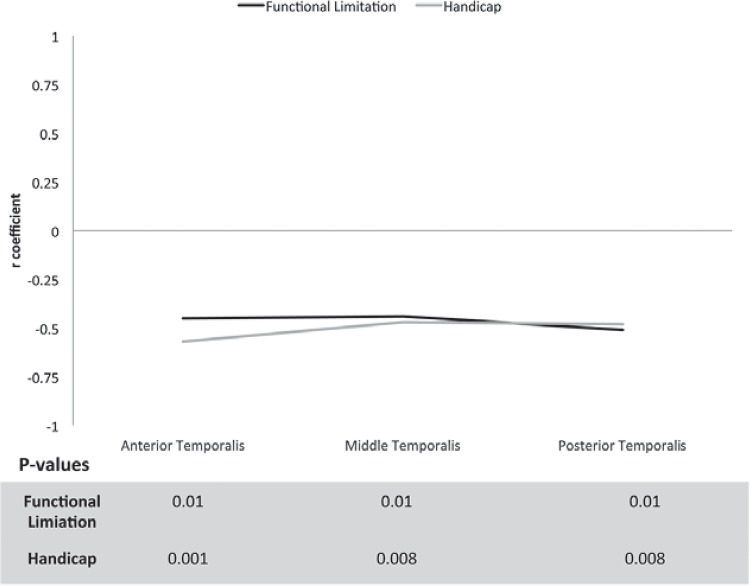
Line chart showing the correlation coefficients between the functional limitation and handicap dimensions of the Oral Health Impact Profile and the pressure pain threshold (PPT) of the anterior, middle and posterior temporalis

A significant negative correlation of moderate magnitude was found between the OHIP (total score) and the PPT values of the anterior temporalis (r=-0.50, 95% CI -0.73 to -0.17, p=0.005) after performing the Bonferroni correction (Figure 2). Moreover, the functional limitation dimension presented significant negative correlations of moderate magnitude with the PPT values for the anterior (r=-0.45, 95% CI -0.71 to -0.09, p=0.01), middle (r=-0.44, 95% CI -0.70 to -0.08, p=0.01), and posterior temporalis muscle (r=-0.51, 95% CI -0.74 to -0.16, p=0.01) (Figure 3). Likewise, the handicap dimension presented significant negative correlations of moderate magnitude with the PPT values for the anterior (r=-0.57, 95% CI -0.77 to -0.26, p=0.001), middle (r=-0.47, 95% CI -0.71 to -0.13, p=0.008), and posterior temporalis muscle (r=-0.48, 95% CI -0.72 to -0.13, p=0.008) (Figure 3).

## DISCUSSION

This cross-sectional pilot study evaluated the correlation between deep pain sensitivity of masticatory muscles and prosthetic aspects, and also between the former factor and the oral-related quality of life of complete denture wearers. The main findings were: a) there is no correlation between masticatory muscle sensitivity and prosthetic aspects; b) the greater the temporalis muscle sensitivity, the poorer the OHRQoL.

Our sample was composed of subjects without any ongoing TMD, presenting no complaints of masticatory myofascial or TMJ pain. There is no consensus on TMD prevalence or incidence either in the elderly population or in complete denture wearers. However, a recently published cross-sectional study that adopted the Research Diagnostic Criteria for Temporomandibular Disorders (RDC/TMD) showed similar prevalence figures for the various types of TMD in Brazilian complete denture wearers: myofascial pain (16.3%), disc displacements (10.8%), and arthralgia, osteoarthritis and osteoarthrosis (19.5%)^19^. Furthermore, findings of low impact on OHRQoL related to facial pain were similar to the evidence revealed in previous studies^6^.

Muscle pain in the elderly population could be associated with the effects of aging and also with weak and impaired muscle activity, resulting from the long-term use of complete dentures^8^ ^,^ ^15^. In fact, pain in the masseter area or masticatory muscle pain on palpation has been associated with the use of complete dentures^22^. Moreover, among the collection of signs and symptoms of TMD, muscle pain could be considered a risk factor for TMD onset^23^. Indeed, epidemiological studies have shown that low PPT values were significantly associated with a higher prevalence of chronic TMD, and could be considered a predictor of TMD incidence^23^. Furthermore, the lower PPT values of the first-onset of TMD cases represented a significant predictor that TMD could become persistent^24^. For this reason, measuring PPT values in the TMD-free subjects is valuable in terms of devising preventive strategies or performing risk analyses.

No relationship was found between PPT and prosthetic factors. This is in line with the current concept that considers the multifactorial etiology of muscle pain, particularly masticatory myofascial pain, since mechanical and occlusal aspects seem to play a minor role in TMD development^16^. The evidence supporting the association of prosthetic factors with signs and symptoms of TMD is tenuous, and there is no association of these factors with TMD diagnosis^19^ ^,^ ^22^. Nonetheless, there was a trend in the correlation between PPT of the anterior temporalis and tooth wear. This could be related to the weakening of jaw muscles, or an impaired muscle function associated with the use of complete dentures^7^.

The influence of emotions and psychological aspects on clinical and experimental pain is well-established^11^. In particular, stress periods are associated with decreased PPT values in masticatory myofascial pain patients^27^. Moreover, depression symptoms and catastrophic thoughts could partially explain the PPT variation related to neck muscles^29^. In line with these concepts, a patient’s perception of oral health also bears implications on pain symptomatology. A study similar to ours showed that a high degree of pressure pain sensitivity is correlated with a poor quality of life in the working population^4^. Our results also support the biopsychosocial model of pain, considering that low PPT values of the anterior temporalis were associated with a poor OHRQoL. The muscle of the temporalis had a bearing on the results of all the correlations. This could be explained by the microstructural differences between the masseter and the temporalis. The latter is mainly responsible for moving or stabilizing the mandible, whereas the former is a power muscle with high fatigue resistance^26^. Lastly, it is important to note that the PPT could be influenced by other factors, such as metabolism, lifestyle, and aging^15^ ^,^ ^30^. The effect of aging on PPT is controversial; however, evidence related to the temporalis muscle showed that PPT increases with age, thus reinforcing the significance of our results^13^.

The correlations between functional limitation and handicap domains with the PPT values of the muscle of the temporalis were also an interesting finding. The subscale analysis showed divergent results in a TMD population, in which the mainly affected dimensions were psychological discomfort and disability^10^. These differences could be explained by the particularities of this study sample (elderly and edentulous). Moreover, we cannot rule out the potential response bias related to unspecific facial pain symptoms not necessarily related to TMD pain, which could affect this population^12^. Although the internal consistency of the OHIP is high (Cronbach’s alpha = 0.963)^18^, it is important to note the psychometric properties of the OHIP are characteristics of the instrument in a specific population and not of the instrument itself. That said, the use of instruments originally designed for the elderly population, e.g., the Geriatric Oral Health Assessment Index (GOHAI)^9^, may be more adequate. Therefore, more research is needed to explore the relationship between PPT and OHRQoL in the elderly in greater detail.

This study offers some insights concerning the relationship between prosthetic aspects and masticatory muscles, and between quality of life of complete denture wearers and masticatory muscles. However, some major limitations should be pointed out: 1) the lack of a control group of dentate patients and of patient self-assessment of denture quality, which makes the drawing of convincing conclusions unsubstantial and precludes the control of confounding factors; 2) there is a possibility of examiner bias associated with the subjective methods used to evaluate the quality of dentures and of the VDO. Although the present study followed guidelines taken from the evidence of other studies^3^, the authors recognize the paucity of criteria with high enough values of validity and reliability to assess the prosthetic aspects of complete dentures.

## CONCLUSION

The deep pain sensitivity of masticatory muscles in complete dentures wearers is associated with OHRQoL, but not with prosthetic factors. However, considering the preventive and prognostic importance of PPT and its possible predictors in complete denture wearers, sound conclusions still need to be confirmed and future research is warranted to endorse the results of this pilot study.
